# Cholera in Jamaica

**Published:** 1855-12

**Authors:** 


### CHOLERA IN JAMAICA.

A copy of the *Falmouth Post and Jamaica General Advertiser*, for October 20, 1855, has been sent us, from which we learn that cholera had again visited this island. The editor of the *Post* passes severe strictures on the medical men for having sanctioned the landing of cholera patients at Kingston from the *Atrato.* It seems that this vessel reached the island, having had several cases of cholera on board, one of which had been fatal two days before. The medical officer of health ordered the ship into quarantine, but the governor on the advice of four medical men liberated the crew. The result was the occurrence on the following day of numerous cases of the disease.

